# Localization and Orientation of Xanthophylls in a Lipid Bilayer

**DOI:** 10.1038/s41598-017-10183-7

**Published:** 2017-08-29

**Authors:** Wojciech Grudzinski, Lukasz Nierzwicki, Renata Welc, Emilia Reszczynska, Rafal Luchowski, Jacek Czub, Wieslaw I. Gruszecki

**Affiliations:** 1 Department of Biophysics, Institute of Physics, Maria Curie-Sklodowska University, 20-031 Lublin, Poland; 2 Department of Physical Chemistry, Gdansk University of Technology, Narutowicza 11/12, 80-233 Gdansk, Poland

## Abstract

Xanthophylls (polar carotenoids) play diverse biological roles, among which are modulation of the physical properties of lipid membranes and protection of biomembranes against oxidative damage. Molecular mechanisms underlying these functions are intimately related to the localization and orientation of xanthophyll molecules in lipid membranes. In the present work, we address the problem of localization and orientation of two xanthophylls present in the photosynthetic apparatus of plants and in the retina of the human eye, zeaxanthin and lutein, in a single lipid bilayer membrane formed with dimyristoylphosphatidylcholine. By using fluorescence microscopic analysis and Raman imaging of giant unilamellar vesicles, as well as molecular dynamics simulations, we show that lutein and zeaxanthin adopt a very similar transmembrane orientation within a lipid membrane. In experimental and computational approach, the average tilt angle of xanthophylls relative to the membrane normal is independently found to be ~40 deg, and results from hydrophobic mismatch between the membrane thickness and the distance between the terminal hydroxyl groups of the xanthophylls. Consequences of such a localization and orientation for biological activity of xanthophylls are discussed.

## Introduction

**Figure 1:**
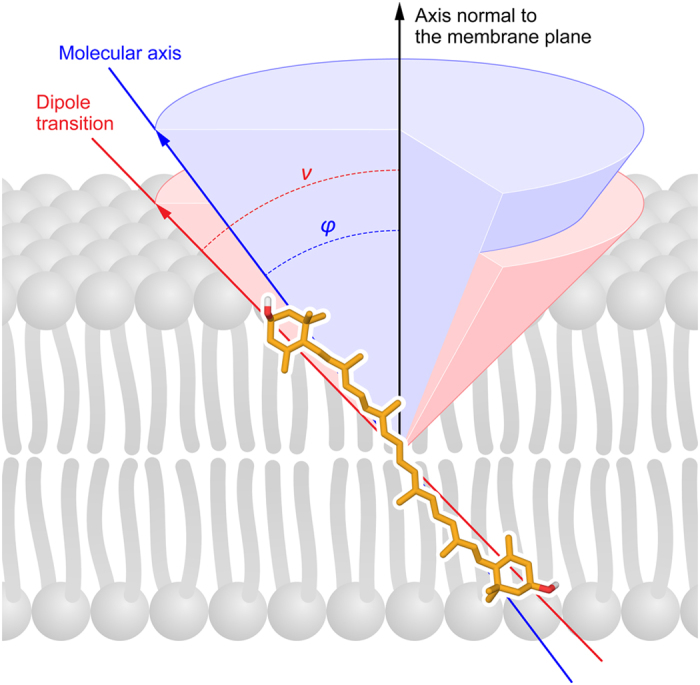
A scheme presenting localization of the molecule of zeaxanthin in the lipid bilayer. Orientation of the axis normal to the plane of the membrane, molecular axis and the transition dipole are marked. The molecular axis has been defined by the linear part of the conjugated C = C double bound system.

Carotenoids, including polar xanthophylls, are ubiquitous in nature and play numerous physiological roles, among which are modulation of the physical properties of lipid membranes and protection against oxidative damage of membrane lipids^1–3^. Localization, organization and orientation of xanthophyll molecules within the lipid bilayers (see the model presented in Fig. 1) are tightly associated with molecular mechanisms responsible for physiological activity of carotenoids in biomembranes and therefore such problems were frequently addressed in the past, in advanced studies^4–7^. In order to precisely determine the orientation of a carotenoid chromophore within the lipid membrane, based on linear dichroism analysis, it was necessary to use relatively high pigment concentrations with respect to lipids or to study model systems composed of many dozens of stacked lipid bilayers^6–9^. Unfortunately, high concentrations result in at least partial aggregation of xanthophylls within the lipid phase^9, 10^, which may considerably affect the accuracy to which the orientation of individual molecules can be determined. Moreover, a model system composed of stacked lipid bilayers seems not to be well suited for precise studies due to the fact that a certain fraction of analyzed molecules may locate in the intermembrane spaces, where it likely shows different orientation behavior compared to the membrane-bound fraction. Indeed, the average orientation angle determined for xanthophylls in the lipid multibilayer systems was found to depend on a number of stacked lipid bilayers in the samples^8^. In the present work we readdress the problem of localization and orientation of xanthophylls within lipid membranes, with application of the experimental and computational approaches which allow to analyze a single lipid bilayer membrane. Two xanthophylls were analyzed, zeaxanthin (Zea) and lutein (Lut), which play biological roles both in the plant and animal kingdoms^1, 11^ (see Supplementary information Fig. S1 for chemical structures). Zea and Lut are particularly interesting from the standpoint of their physiological activity in the *macula lutea* in the retina of the human eye^12, 13^. Both Lut and Zea are dietary carotenoids which are selectively accumulated in the human eye retina and are found to prevent the AMD (age-related macular degeneration) syndrome^13^. The concentration of Zea in the central part of the *macula* is significantly higher than the concentration of Lut, and an opposite proportion is observed in the peripheral retina, suggesting differences in molecular mechanisms of biological activity of those xanthophylls in the eye^14^. Macular xanthophylls are distributed between the lipid and protein components of the membranes^13^ and unveiling of determinants of localization, molecular organization and orientation of xanthophylls in biomembranes seems necessary to understand molecular mechanisms associated with the physiological activity of xanthophylls in the human eye. Importantly, Zea is a xanthophyll that plays a vital physiological function in protection of the photosynthetic apparatus of plants against strong-light-induced damage^15^. The photosynthetically-active xanthophylls play their biological roles embedded in a protein environment of the pigment-protein complexes. On the other hand, involvement of Zea in the reactions of the xanthophyll cycle makes this pigment at least temporarily present and functional directly in the lipid phase of the thylakoid membranes^16–18^. Owing to this fact, localization and orientation of Zea in lipid bilayers is also a problem interesting and important from the standpoint of photosynthesis research.

## Results

### Spectroscopic analysis of xanthophyll-containing membranes

**Figure 2:**
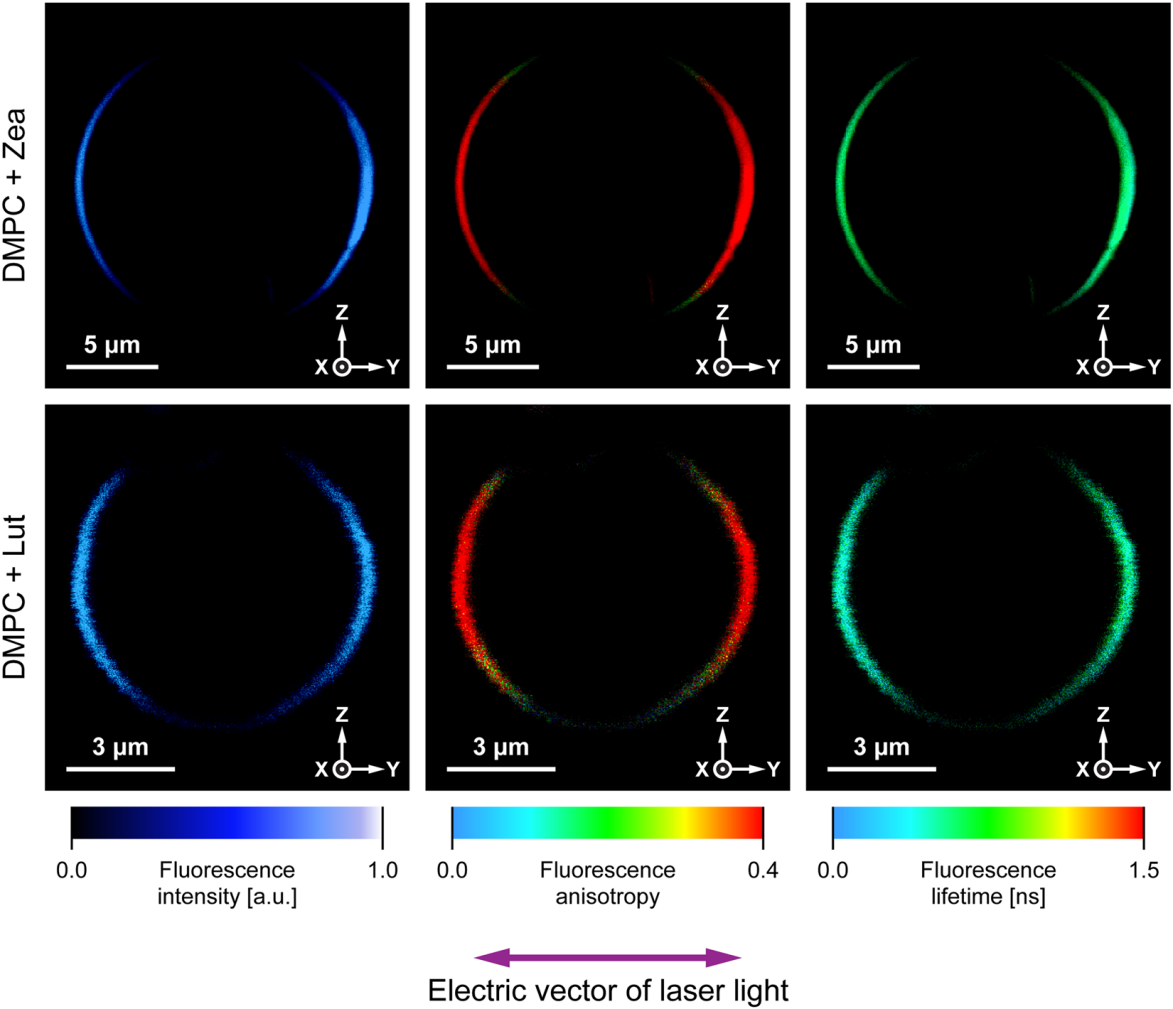
The results of microscopic imaging of single lipid vesicles containing xanthophylls incorporated to the lipid phase. Three panels show fluorescence intensity, anisotropy and lifetime. The images represent an equatorial vesicle cross-section in the focal plane of a microscope. The upper panel presents a liposome containing incorporated zeaxanthin, the lower panel presents a liposome containing incorporated lutein. The direction of the electric vector of probing light is shown below the images (collinear with the Y axis).

Figure 2 presents fluorescence microscopy images of single giant unilamellar vesicle (GUV) structures containing Zea or Lut. The fact that both xanthophylls were incorporated into the membranes at relatively low concentration of 0.5 mol% with respect to lipids assures monomeric organization of their molecules in the lipid phase. Quantitative analysis of fluorescence intensity distribution in the microscopic images representing the equatorial cross-sections of giant unilamellar liposomes enables precise determination of orientation of transition dipoles of fluorescing molecules with respect to the axis normal to the membrane plane^19^. In the case of polyenes, one can assume collinearity of the transition dipoles for light absorption and emission and an angle of ~10 ÷ 15 deg between the dipole transition and the molecular axis defined by the direction of the conjugated double bond chain^20^ (see Fig. 1). Due to the photoselection rule, the molecules with the transition dipoles oriented parallel to the membrane plane (“horizontal” orientation) will give rise to fluorescence signal in the upper and lower portions of the liposome cross-section, while the molecules with the transition dipoles oriented perpendicularly with respect to the membrane plane (“vertical” orientation) will give rise to fluorescence signal in the left-hand and right-hand sectors of the liposome^19^. The results of the experiments show that photoselection is also very clearly pronounced in the images based on fluorescence anisotropy^19^. Relatively high fluorescence anisotropy levels, manifested by red color according to the color coded scale, in the left-hand and right-hand sectors of the liposomes, both in the case of Lut and Zea, indicates roughly vertical orientation of both xanthophylls in the membrane (the average orientation angles lower than the magic angle of 54.7 deg). Table 1 presents the results of the determinations of the xanthophyll orientation, according to the approach based on integration of fluorescence intensity in selected cross-sections of the pigment-containing GUV^19^. As can be seen, the orientation angles of Zea (42.7 ± 0.9) and Lut (41.8 ± 1.4), determined in a single lipid bilayer (the angle ν on Fig. 1), are very close to each other. The apparent experimental uncertainty levels were found to be relatively low (S.D. ~1 deg). However, it has to be emphasized that the experimental result represents an averaged value of potentially differently oriented pools of molecules.

**Table 1:** Averaged orientation angle between the axis normal to the plane of the lipid bilayer formed with DMPC and the transition dipole or the molecular axis of a polyene chain of lutein and zeaxanthin present in the membrane.

Orientation angle	Lutein [deg]	Zeaxanthin [deg]
Transition dipole (ν)	41.8 ± 1.4	42.7 ± 0.9
Polyene axis (φ)	36.8 ± 1.7	36.5 ± 1.8

The data on orientation of the dipole transitions are based on the fluorescence-detected linear dichroism experiments (n = 9 liposomes from 3 independent preparations ± S.D.) and the data on orientation of molecular axis are based on the molecular dynamics simulations (standard error of the means obtained from uncertainties in the free energy profiles in Fig. 4C).

**Figure 3:**
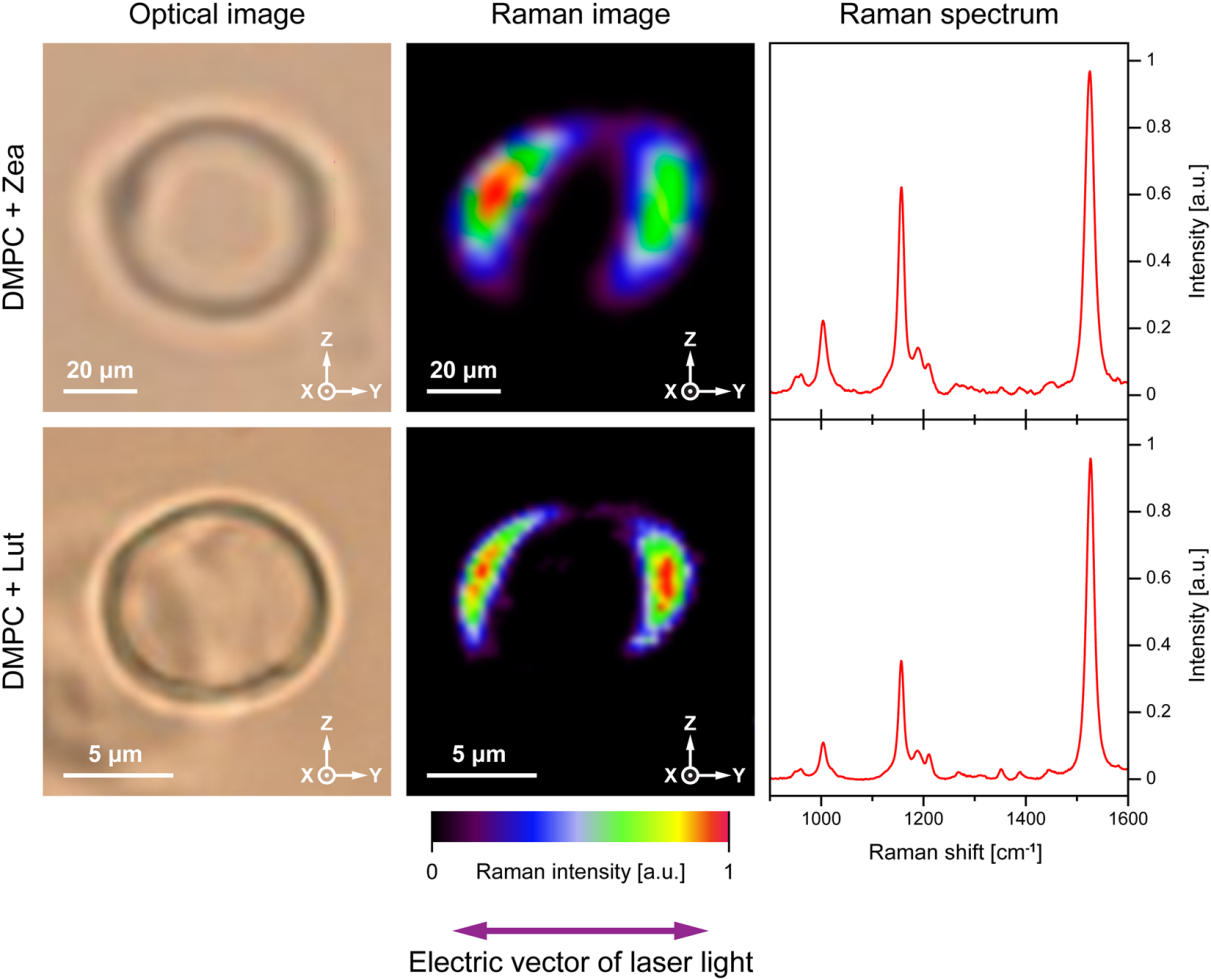
The results of Raman microscopic imaging of single lipid vesicles containing xanthophylls incorporated to the lipid phase. Three panels show optical images, Raman images and averaged Raman scattering spectra recorded during imaging. The images represent an equatorial vesicle cross-section in the focal plane of a microscope. The upper panel presents a liposome containing incorporated zeaxanthin, the lower panel presents a liposome containing incorporated lutein. The images are based on the integration of the spectra in the range 1500–1550 cm^−1^. The direction of the electric vector of probing light is shown below the images (collinear with the Y axis).

Figure 3 presents the exemplary results of the Raman microscopy imaging of the xanthophyll-containing unilamellar liposomes. As can be seen, both in the case of Lut- and Zea-containing membranes, the imaging based on a resonance Raman spectroscopy shows selectively the pigment molecules located in the left-hand and in the right-hand sectors of the liposomes. The reason of such an effect is photoselection. All the pigment molecules embedded in the liposomes give rise to Raman spectra but this is especially the case for the molecules whose transition dipoles of the electronic transitions are collinear with the electric vector of a laser beam, owing to coupling with the electronic transition (a resonance effect)^6^. Such a clear effect supports very strongly the conclusions based on the fluorescence microscopy analysis, above. The resonance Raman spectra of the xanthophylls, collected during the liposome imaging (see the right-hand panels, Fig. 3), demonstrate appearance of a single spectral form, which is a direct indication of monomeric organization of both Lut and Zea in the lipid phase, at the relatively low xanthophyll concentration applied (0.5 mol% with respect to lipid).

### Molecular dynamics simulations of membrane-embedded xanthophylls

**Figure 4:**
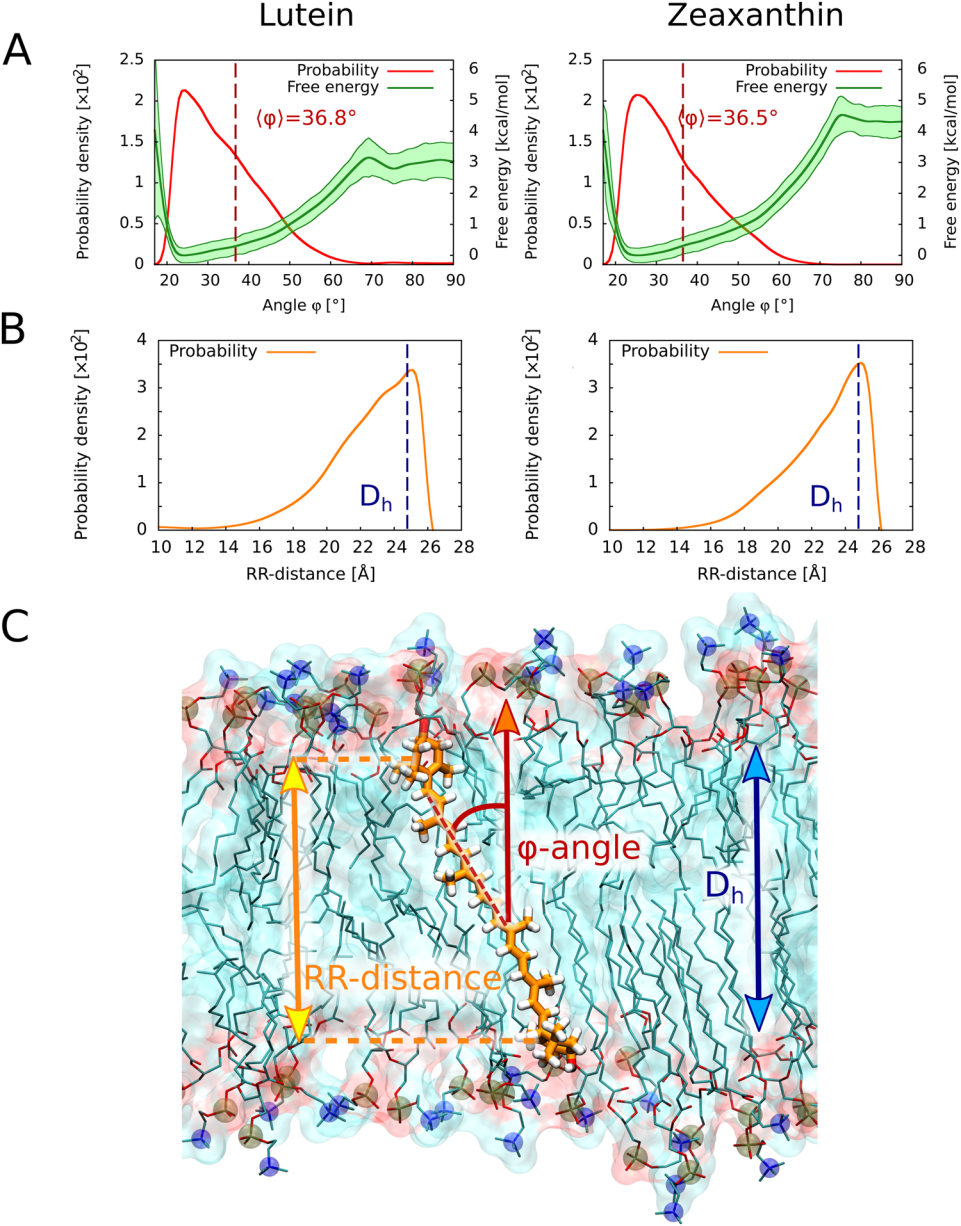
Results of molecular dynamics simulations of xanthophylls embedded in the lipid bilayer. (**A**) Free energy profiles (green) and corresponding probability distributions (red) of the angle between the axis of the polyene chromophore of lutein (left) or zeaxanthin (right) and the membrane normal (φ-angle). The vertical dashed lines show the equilibrium averages of φ-angle obtained from the calculated distributions. (**B**) Probability distributions of the distance between the terminal rings of both xanthophylls projected on the membrane normal (RR-distance). The vertical dashed lines show the thickness of the hydrophobic core of the membrane (D_h_). (**C**) MD simulation snapshot showing a representative arrangement of a lutein molecule embedded in the DMPC membrane.

To understand structural determinants of the orientation of Lut and Zea in a lipid membrane, we first calculated how the free energy of the system containing a single xanthophyll molecule in a lipid bilayer formed with dimyristoylphosphatidylcholine (DMPC) depends on the angle between the axis of the polyene chromophore and the membrane normal (φ-angle in Fig. 1). To this end, we used umbrella-sampling molecular dynamics simulations (see Methods). The resulting free energy profiles with the well-pronounced minima around 25 deg and the corresponding φ-angle distributions shown in Fig. 4A confirm that both xanthophylls orient vertically in a DMPC bilayer, consistently with the previous molecular dynamics study^21^. In fact, the average orientation angles obtained from the φ-angle distributions in Fig. 4A are equal to 36.5 ± 1.7 and 36.8 ± 1.8 deg for Zea and Lut, respectively, showing a very good agreement with our experimental data. Notably, the difference between the experimental and computational averages is markedly smaller than the deviation of the transition dipole from the axis of the chromophore (ν-angle in Fig. 1). As can be also seen from Fig. 4A, the distributions of φ-angle obtained for Zea and Lut xanthophylls are very similar, ranging from ~19 to ~60 deg, which can be attributed to a similar structure of both xanthophylls. Interestingly, however, a generally unfavorable horizontal orientation (φ > 70 deg) is by ~1.4 kcal/mol less stable for Zea than for Lut resulting in the equilibrium population of horizontally oriented Lut molecules to be ~exp(1.4/RT) ≈ 10 times greater than that of Zea.

To examine the relation between the structure of the DMPC membrane and the observed orientation of xanthophylls, we computed the distribution of the distance between the terminal rings of the molecules projected on the membrane normal (RR-distance). As shown in Fig. 4B, the most probable RR-distance of 25 Å, for which both rings are located at the opposite boundaries between the polar and hydrophobic regions of the bilayer (Fig. 4C), coincides with the thickness of the hydrophobic part of the DMPC membrane (D_h_ = 24.8 Å, see Fig. S2 for definition and details). This strongly suggests that the observed tilted arrangement of the xanthophylls (Fig. 4C) arises from the hydrophobic mismatch between the length of the xanthophyll molecules and the hydrophobic thickness of the liquid-crystalline DMPC bilayer. More perpendicular orientation with respect to the membrane plane leads to exposure of the hydrophobic rings to the polar region of the bilayer and to water phase, which is highly unfavorable, as seen from a steep increase of the free energy for φ-angles smaller than ~20 deg in Fig. 4A.

When located at the boundary of the hydrophobic membrane core, the rings of xanthophylls may form hydrogen bonds with polar headgroups of DMPC. To investigate if hydrogen bonding patterns contribute to the observed preference for vertical arrangement, we calculated the probability of hydrogen bond formation between the xanthophyll hydroxyl groups and the carbonyl and phosphate groups of lipids. The results presented in Fig. S3 show that upon reorientation to horizontal arrangement Lut and Zea lose 33 and 26% of hydrogen bonds, respectively. This suggests that the vertical orientation is at least partially stabilized by hydrogen bonds with the polar region of the membrane. Since the ε-ring of lutein is allowed to rotate around the axis of the chromophore (see Fig. S4), in its horizontal orientation, lutein can more flexibly incorporate into the polar region of the membrane than it is the case for the stiffer zeaxanthin with conjugated β-rings (Fig. S5). More efficient interactions of the lutein hydroxyl groups with DMPC polar headgroups and water may in turn explain its noticeably higher capacity to align parallel to the membrane surface and provide a previously observed kinetic stability of the horizontal orientation ranging up to tens of nanoseconds^22^.

## Discussion

Comparison of the orientation angle of the transition dipole of Zea, found in the present study, with the values reported previously^8^, shows that the angles determined in a single lipid bilayer formed with DMPC are larger than in the lipid multi-bilayer model systems composed of the same lipid: ~43 deg versus ~25 deg^8^ The fact that in the earlier work orientation angles were found to depend on a number of stacked lipid bilayers^8^ suggests that pools of the pigment molecules located in the adjacent membrane interface could influence determination of an orientation angle of the xanthophylls present in the lipid phase. Moreover, in the present study, the xanthophyll concentration was at a very low level (0.5 mol% with respect to lipid), in order to assure monomeric organization of pigment molecules embedded in the membranes. It is very likely that different molecular organization forms (dimers, tetramers, bulk aggregates) adopt different orientation in a lipid bilayer. This is, most probably, also a major cause of differences between the results presented in this work and reported previously, based on determinations in lipid multi-bilayer systems. Interestingly, in the present study we did not observe any significant differences between the orientation angles determined for Zea and Lut, in contrast to the previous studies carried out in a lipid multi-bilayer system^23^. In those studies the transition dipoles of Lut were found to form greater angles with respect to the axis normal to the membrane, as compared to Zea under the same conditions^24^. The fact that such differences are not observed in the experimental system composed of a single lipid bilayer implies that molecules of Lut exhibit a higher affinity to be localized in the inter-membrane region, as compared to Zea. This could be related to the fact that the double bond present in one of the terminal rings of Lut (ε ring), in the C4′ = C5′ position, is not conjugated with the polyene chain of the pigment. As shown by our quantum mechanical calculations, this allows for the rotation of the entire ε-ring about the C6–C7 single bond, which is not possible in the case of two terminal β-rings of Zea, due to the fact that both double bounds, C5 = C6 and C5′ = C6′ are conjugated with the polyene chain. In our opinion, such a specific ability of Lut to occupy an intermembrane space between the adjacent lipid membranes may be potentially physiologically relevant, e.g. in the neural cells of the brain^25^.

Another interesting issue is directly related to the differences between the results of the experimental and computational studies presented in this work. The fluorescence-detected linear dichroism measurements enabled determination of the average orientation angles between the transition dipoles of the main electronic transition of Zea and Lut and the axis normal to the membrane plane. On the other hand, molecular dynamics simulations allowed us to determine the distribution of possible orientations of molecular axes of the xanthophylls in the membrane. According to the theory, the transition dipole of a linear polyene composed of 11 conjugated C = C bonds (e.g. lycopene) forms an angle of ca. 15 deg with the polyene axis^20^. The picture is a bit more complicated in the case of pigments in which conjugation extends to the terminal rings (such as Zea), due to the fact that the directions of the terminal double bonds are tilted with respect to the central, linear polyene chain^26^. Moreover, the entire conjugated double bond system becomes twisted and consequently slightly S-shaped (see Fig. 1). Comparison of the experimental and computational data presented in Table 1 lead us to conclude that the transition dipoles are tilted with respect to the main molecular axes by ca. 5 deg and 6 deg for Lut and Zea respectively.

As shown by our molecular dynamics simulations, the vertical arrangement of both the xanthophylls is stabilized by the formation of hydrogen bonds between the terminal hydroxyl groups located in the C3 and C3′ positions with lipid polar headgroups which constitute the two opposite polar membrane regions. We also found that the observed tilted orientation of the xanthophylls results from the hydrophobic mismatch between the length of these molecules and the hydrophobic thickness of the membrane. This means, that the xanthophyll orientation can be predicted by comparison of the distance between the terminal hydroxyl groups and the thickness of the hydrophobic core of the membrane. Such a transmembrane location of polar carotenoids provides very good conditions for interaction of a rigid polyene chain with alkyl chains of lipids, via van der Waals interactions. This type of interaction is considered as an important activity of xanthophylls having a consequence in modification of the physical properties of lipid membranes^2^. Macular xanthophylls are predominantly accumulated in the axons forming the Henle fiber layer located in the frontal (inner) part of the retina^13^. Such a localization of chromophores reduces light transmission, protecting photoreceptors against photo-damage. A roughly vertical orientation of the transition dipoles of Zea and Lut, with the respect of the axis normal to the membrane plane, reported in the present work, provides good conditions for the macular xanthophylls to act as a blue light filter. This is owing to the fact that direction of the light beam penetrating the inner layers of the retina, will form a small angle with the direction of the axons and therefore the electric vector of electromagnetic radiation (perpendicular to the direction of light propagation) will have a nonzero component in the direction of the xanthophyll transition dipoles. Importantly, such an organization of the xanthophyll-lipid membranes has also a direct effect on reducing penetration of molecular oxygen into the hydrophobic membrane core, thus protecting vulnerable membrane regions against oxidative damage^14^. Such a mechanism, combined with neutralization of reactive oxygen species, can be considered as an important molecular determinant of the pronounced photoprotective activity of Zea with respect to the thylakoid membrane lipids^27, 28^. In contrast to previous studies, no differences between the localization and orientation of lutein and zeaxanthin has been observed. This suggests that other factors than a behavior of xanthophyll molecules in a lipid phase of biomembranes are associated with the fact that both lutein and zeaxanthin are accumulated and differently distributed in the human eye retina. Among such factors may be, for example, interaction with the specific, xanthophyll binding proteins.

In conclusion, in this work we present a combined experimental and computational study on localization and orientation of two polar carotenoids, lutein and zeaxanthin, in a single lipid bilayer formed with dimyristoylphosphatidylcholine. The results let conclude that both xanthophylls adopt a transmembrane localization, stabilized by formation of hydrogen bonds between the terminal hydroxyl groups of xanthophylls and the opposite polar regions of the lipid bilayer. The pigment orientation was concluded to be determined by the distance between the terminal polar groups of xanthophylls and the thickness of the hydrophobic core of the membrane. Such localization and orientation of both xanthophylls is an important structural determinant of their high effectiveness in stabilization and protection of biomembranes against oxidative damage.

## Methods

### Chemicals

Zeaxanthin ((3R,3′R)-β,β-carotene-3,3′-diol) and lutein ((3R,3′R,6′R)-β,ε-carotene-3,3′-diol) (see the supplementary Fig. S1 for chemical structures) were isolated from plant material and purified chromatographically according to the routine procedures described in detail previously^29^. Lutein was isolated from spinach (*Spinacia oleracea*) leaves and zeaxanthin from fruits of *Lycium barbarum*. L-α-dimyristoylphosphatidylcholine was purchased from Sigma Aldrich (USA).

### Liposome preparation

Giant unilamellar vesicles were formed of DMPC with either lutein or zeaxanthin at 0.5 mol% xanthophyll with respect to lipid. The xanthophyll concentration selected (0.5 mol% with respect to lipid) corresponds directly to the concentration estimated in the macular membranes^30^. Bone and Landrum have estimated a surface density for the pigment of 1 molecule per 62 nm^2^ ^30^, which corresponds to ~0.48 mol%, assuming a limiting area of a single lipid molecule ~0.6 nm^2^ ^31^ and taking into account that one xanthophyll molecule spans a lipid membrane formed by two monomolecular layers. Following such an estimation, the concentration of macular xanthophylls has been concluded to be lower than 1 mol% in the outer photoreceptor membranes^32, 33^. Taking into consideration fact that xanthophylls present in the photosynthetic membranes are bound to the functional pigment-protein complexes and only the pigments involved in the xanthophyll cycle can be directly present in the thylakoid membrane lipid phase, one can expect similarly low xanthophyll concentration in the lipid bilayers of the photosynthetic apparatus of plants^34^. Under certain conditions (e.g. plants acclimated to higher light intensities) a fraction of the xanthophyll cycle pigments present directly in the thylakoid membrane lipid phase can be higher^34^. On the other hand, based on the fact that in order to be solubilized in the lipid phase, 1 molecule of violaxanthin requires minimum 20 molecules of MGDG (monogalactosyldiacylglycerol) or 100 molecules of DGDG (digalactosyldiacylglycerol)^34^, one can estimate that a fraction of xanthophylls in the lipid phase of the thylakoid membranes does not exceed 1–5 mol% (depending on local homogeneity of the membrane). Final lipid concentration in a water phase was 29 mM. In order to prepare liposomes containing xanthophylls, a pigment was added to a lipid solution in ethanol. Constituents of a lipid phase of liposomes were deposited to two platinum electrodes (35 × 4 × 0.5 mm) by means of evaporation from a solution, under a stream of gaseous nitrogen. Residuals of organic solvents were removed during incubation for 1 h under vacuum. The two platinum electrodes, covered by deposited lipid films, were fixed in the Teflon holder at a distance of 5 mm. Electrodes were placed in a cuvette containing buffer solution (1.4 mL, 20 mM Tricine, 10 mM KCl, pH 7.4). Electric connections were attached to an AC field supply (DF 1641A). GUV electroformation was carried out over 2 h with an applied AC sinusoidal field with 10 Hz frequency and voltage 3 V (peak-to-peak), according to the recommendations from the literature^35^. The temperature during electroformation was stabilized at 25 °C (above the main phase transition of membranes formed with DMPC, ~23 °C).

### Fluorescence microscopy analysis

The experiments were performed on a two-channel MicroTime 200 (Picoquant, Germany) confocal system coupled to Olympus IX71 inverted microscope. The instrument possessed objective piezo-scanner with 80 µm × 80 μm imaging range at nominal 1 nm positioning accuracy. A sample was illuminated with 470 nm pulsed laser focused on interesting objects by water immersed objective (Olympus Plan Apo NA = 1.2, 60×). The laser power was 0.4 µW. The observation was made using dichroic (ZT 473 rdcxt from Analysentechik) and 520 nm band-pass filter (FF01-520/35 from Semrock). The confocal pinhole was used 50 μm in diameter. Fluorescence beam was split by polarizing cube and observed by two orthogonally polarized analyzers (Single Photon Avalanche Diodes). The perpendicular (I_VH_) and parallel (I_VV_) intensities were measured and further used to calculate the anisotropy as defined:

1 $$r=\frac{{I}_{VV}-G{I}_{VH}}{{I}_{VV}+2G{I}_{VH}}$$

First subscript of the emission intensities indicate the direction of polarization of the exciting light with respect to the laboratory frame of reference (V-vertical), and the second subscript indicates direction of polarization of the emitted light with respect to the laboratory frame of reference (V-vertical, H-horizontal). G was an instrumental correction factor (in our case equal 0.99) and was evaluated in separate measurements on a long fluorescence lifetime fluorophore.

The effective confocal volume V_conf_ of the system was calculated based on the formula referring to its ellipsoidal shape approximation:

2 $${V}_{conf}={(\pi )}^{3/2}{x}_{o}^{2}{z}_{o}$$

Where x_o_ is a lateral and z_o_ the axial radius of the volume. In our configuration alignment x_o_ = 202 nm and z_o_ = 1010 nm. Hence V_conf_ = 0.23 fl.

The microscopy system measured fluorescence anisotropies as well as lifetimes of the sample in the same time. The intensity decays were analyzed in terms of an exponential model using SymPhoTime 64 v. 2.1 software (PicoQuant).

The intensity decays for each sample were fitted with the multi-exponential model:

3 $$I(t)=\sum _{i}{\alpha }_{i}\exp (-t/{\tau }_{i})$$

where τ_i_ are the decay times and α_i_ are the pre-exponential factors (amplitudes) of the individual components (∑α_i_ = 1). The mean decay time (amplitude-weighted lifetime) is given by:

4 $$\langle \tau \rangle =\sum _{i}{\alpha }_{i}{\tau }_{i}$$

At least 10 giant unilamellar vesicles with lutein and 10 vesicles with zeaxanthin, from three different preparations, were imaged and analyzed. Representative images are presented in the paper.

### Determination of the xanthophyll orientation

Orientation of dipole transitions of xanthophylls embedded in a single lipid bilayer was determined according to the approach reported and described in detail previously^19^. An average orientation angle between the direction of the transition dipole of xanthophylls and the axis normal to the plane of the membrane (ν) was determined based on integration of fluorescence signals in the small membrane fragments spanned by the axis Z (F^Up-Down^) and the axis Y (F^Left-Right^) in the image of equatorial cross-section of the giant unilamellar vesicle. The membrane fragments limited by the cones defined by an angle of 10 deg have been selected for integration. Owing to the fact that fluorescence lifetime was homogenously distributed in entire liposomes the orientation angle was calculated according to the reduced formula^19^:

5 $$\frac{{F}^{Up-Down}}{{F}^{Left-Right}}=\frac{1}{2}{\tan }^{2}v$$

### Raman imaging analysis

Raman spectroscopy was carried out using an inVia confocal Raman microscope (Renishaw, UK) with argon laser (Stellar-REN, Modu-Laser™, USA) operating at 457 nm (set at 70 μW power at the sample), equipped with 60x water immersed objective (Olympus Plan Apo NA = 1.2). Optical images of xanthophyll-containing GUV were obtained and elaborated with WiRE 4.1 software (Renishaw, UK). Based on such images, areas of approximately 10 μm × 10 μm for Raman scanning were selected and mapped with 0.5 μm spatial resolution. For the purpose of this study, all the images were recorded with a light intensity as low as possible. At each point of Raman image map the spectra were recorded with about 1 cm^−1^ spectral resolution (2400 lines/mm grating) in spectral region 350–1900 cm^−1^ using EMCCD detection camera Newton 970 from Andor, UK. Images were acquired with use of the Renishaw WiRE 4.1 system at high resolution mapping mode (HR maps). Acquisition time for a single spectrum was 0.1 s. All spectra were pre-processed by cosmic ray removing, noise filtering and baseline correction using WiRE 4.2 software from Renishaw, UK. At least 10 giant unilamellar vesicles with lutein and zeaxanthin were imaged and analyzed. Representative images are presented in the paper.

### Molecular dynamics simulation

The simulated systems, constructed with the CHARMM Membrane Builder^36^, were comprised of a single molecule of lutein or zeaxanthin embedded in a lipid bilayer composed of 110 DMPC molecules, solvated with approximately 3250 water molecules and 10 K^+^ and Cl^−^ ions to provide physiological ionic strength. The CHARMM36 forcefield^37^ was used for xanthophyll and DMPC molecules and TIP3P model^38^ was used for water. The missing torsion potential for the rotation of the ε-ring of lutein was obtained by fitting to the potential energy surface calculated at the MP2/6-31 g(d) level of theory with Gaussian09^39^, using a relaxed scan approach. The scan was performed by incrementing the dihedral angle C1′-C6′-C7′-C8′ (see Fig. S4) in 5° steps from -180° to 180°. Force Field Toolkit^40^ was used for fitting the dihedral parameters to the reference results obtained from the rotational scan. The values of obtained parameters are presented in Table S1 and the relative QM and MM energy profiles for the rotation of the considered bond are compared in Fig. S4. The full set of parameters used for xanthophylls is provided in Tables S2–S4 and in the supplementary text file.

All simulations were carried out using NAMD^41^. The simulations were performed in NPT ensemble with the temperature kept at 320 K by Langevin dynamics and pressure kept at 1 Bar with the Langevin piston method^42^. Particle Mesh Ewald algorithm was used for a long range electrostatics interactions with real-space cutoff of 10 Å^43^. Van der Waals interactions were evaluated using a smooth cutoff of 12 Å with a switching radius of 10 Å. The equations of motion were integrated with the velocity Verlet algorithm with a time step of 2 fs. Prior to the free energy calculations the systems were equilibrated in seven steps according to the procedure described in the CHARMM Membrane Builder, during which the energy of the system was first minimized, and next equilibrated in 5 steps with gradually decreasing restraint forces applied to the membrane components^36^. The umbrella sampling (US) method was used to obtain the free energy changes accompanying the variation of the angle between the axis of the xanthophyll chromophore and the membrane normal (φ-angle in Fig. 1). As a working reaction coordinate, we used the distance between the centers of mass of each half of the conjugated chain projected on the bilayer normal (z-distance), similarly to what has been done in a previous study^21^. The initial configurations for umbrella sampling simulations were obtained from a 40 ns long steered-MD simulations, in which the center of harmonic potential applied to z-distance was moved from 11.8 to 0 Å, which corresponds to the rotation of the xanthophyll molecule from the vertical to horizontal orientation. In the actual US calculations, a harmonic biasing potential with a spring constant equal to 3 kcal mol^−1^ Å^−2^ was used to restrain the system in 10 equally spaced overlapping windows from 0 to 10.8 Å and an additional window at 11.8 Å. For each of these windows, 500 ns molecular dynamics simulation was performed and the data was post-processed using the weighted histogram analysis method (WHAM)^44^. Uncertainties in the free energy profiles were estimated using Monte Carlo bootstrap method accounting for the autocorrelation of the time series. The acquired free energy profiles were then mapped to the angle between the xanthophyll polyene chromophore and the bilayer normal using one-to-one mapping.

## Electronic supplementary material

Supplementary Information

supplementary data
