# Supplementary material for: Localization and Orientation of Xanthophylls in a Lipid Bilayer
Source: Sci Rep. 2017 Aug 29;7:9619. doi: 10.1038/s41598-017-10183-7 (PMC5575131; doi:10.1038/s41598-017-10183-7)
Supplement: Supplementary file 1 — Supplementary Information [file 41598_2017_10183_MOESM1_ESM.pdf]

Supplementary Information

Localization and Orientation of Xanthophylls in a Lipid Bilayer

Wojciech Grudzinski[1], *Łukasz Nierzwicki*[2], *Renata Welc*[1], *Emilia Reszczynska*[1],

Rafał Luchowski[1], *Jacek Czub*[2,*], *Wieslaw I. Gruszecki*[1,*]

1. Department of Biophysics, Institute of Physics, Maria Curie-Skłodowska University, 20-031 Lublin, Poland
2. Department of Physical Chemistry, Gdansk University of Technology, Narutowicza 11/12, 80-233 Gdansk, Poland

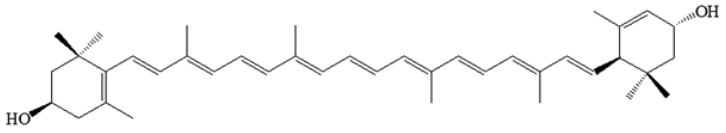

Lutein

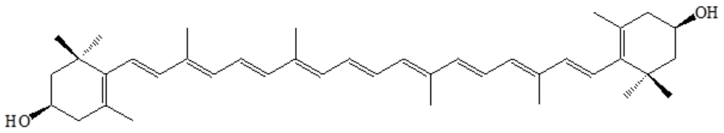

Zeaxanthin

Figure S1 Chemical structure of lutein and zeaxanthin.

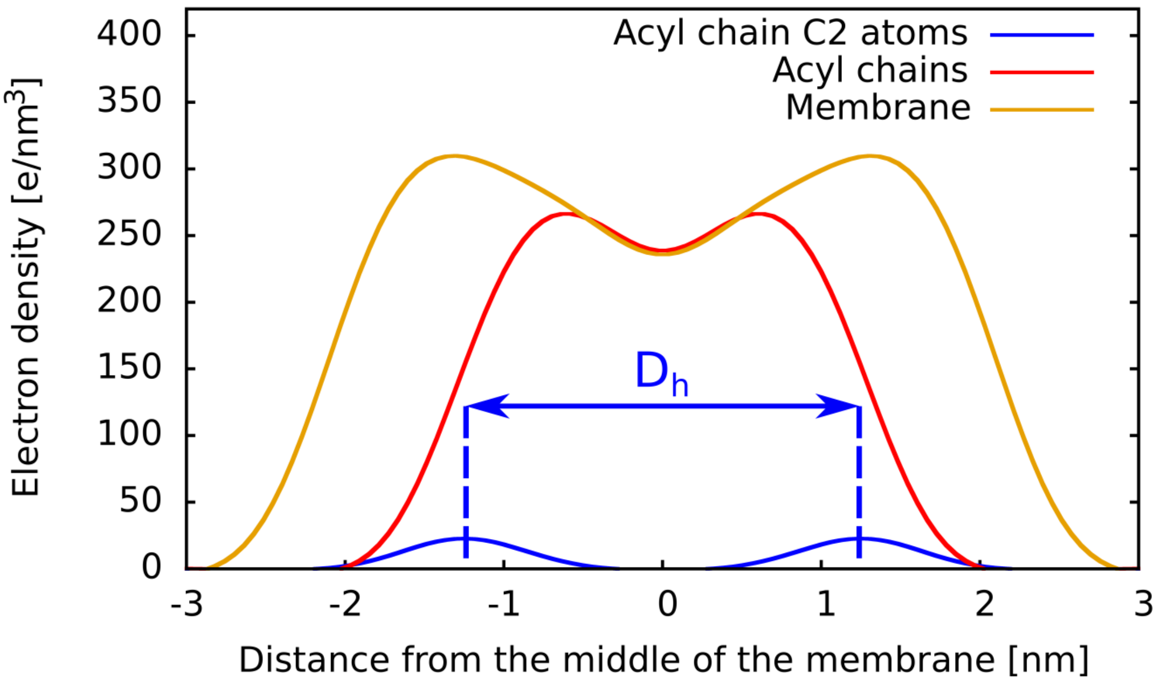

Figure S2. Electron density profiles of the selected components of the simulated DMPC bilayer along the membrane normal. The blue arrow shows the thickness of the membrane hydrophobic core, defined as the distance between the average positions of the acyl chain C2 atoms (the first methylene groups) from the opposite leaflets.

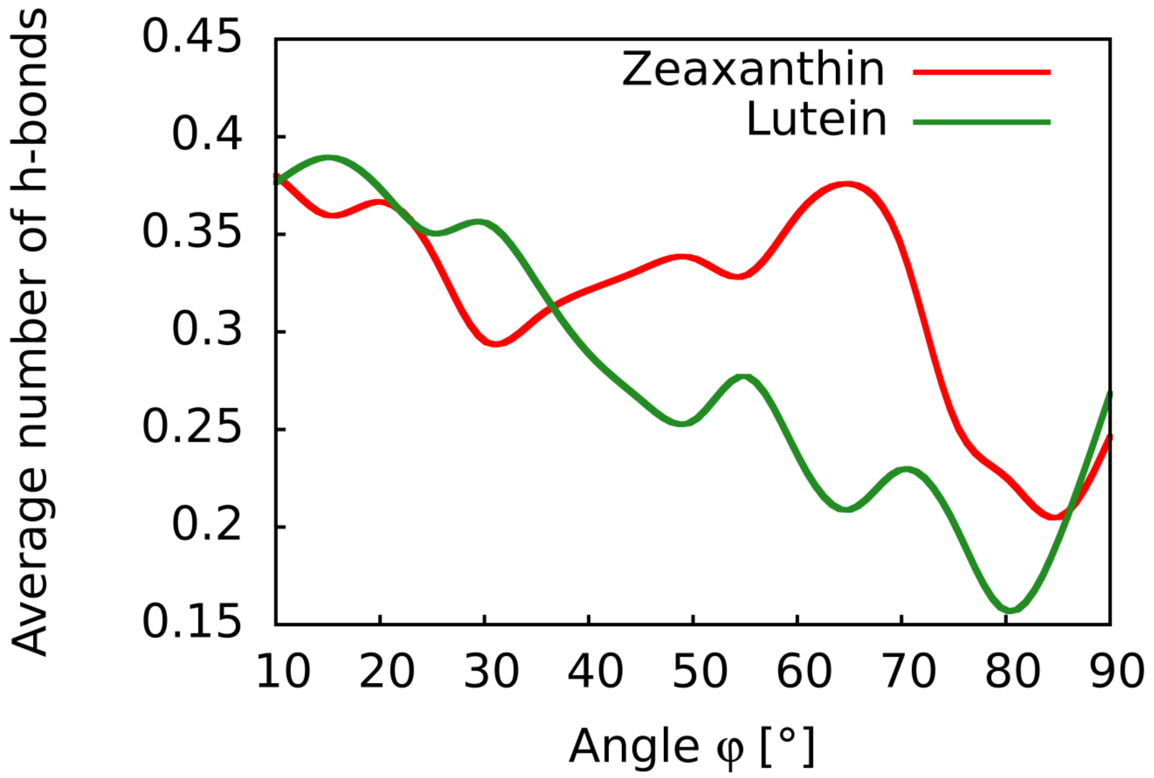

Figure S3. Average number of hydrogen bonds formed by the xanthophyll molecules with the polar headgroups of DMPC as a function of φ-angle.

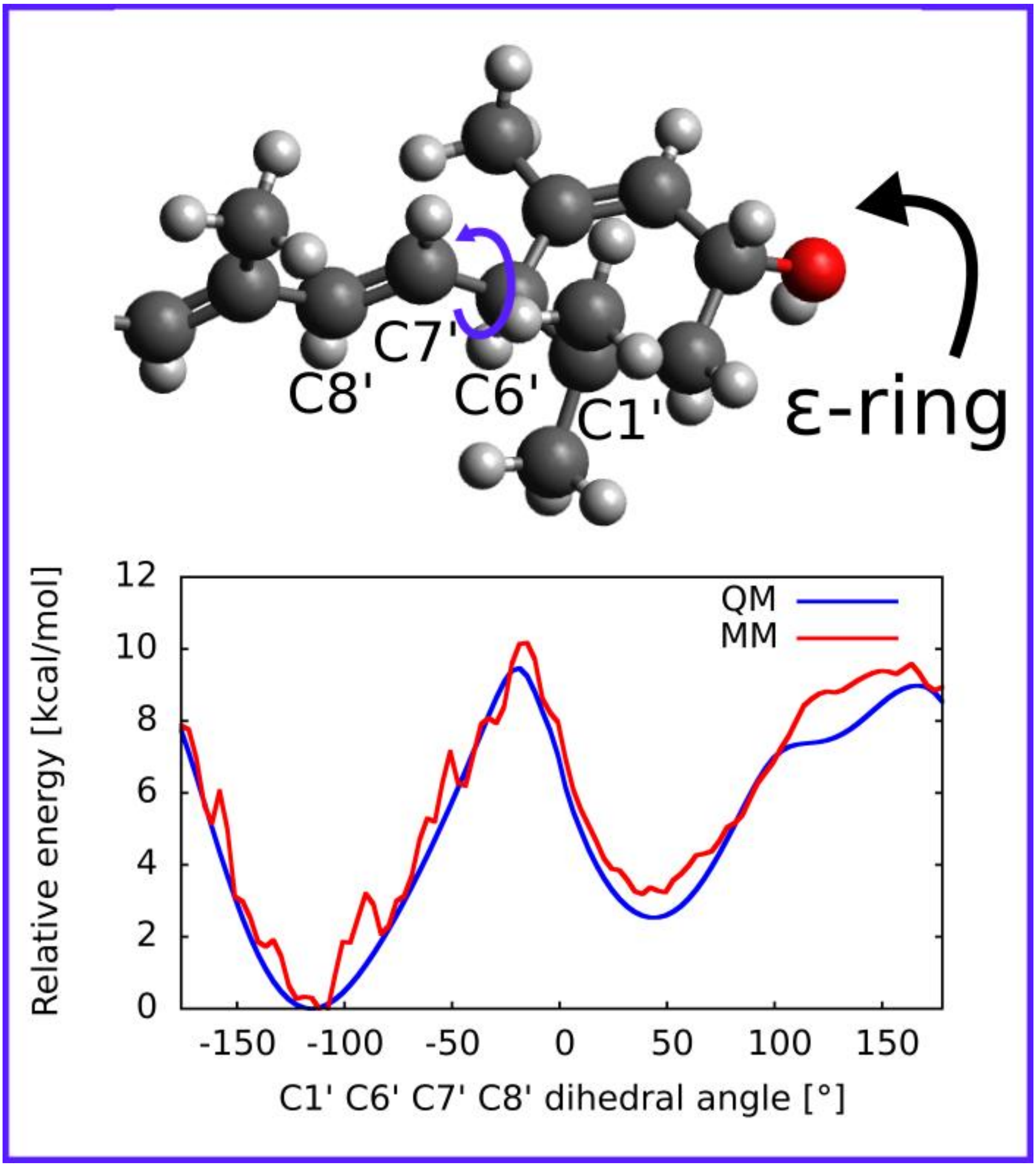

Figure S4. Definition of the dihedral angle C1'-C6'-C7'-C8' corresponding to the rotation of the lutein ε-ring with respect to the polyene chromophore (top) that was used in our quantum mechanical rotational scan (bottom). Fit of the force field energy (red) to a quantum mechanical rotational scan of the dihedral angle C1'-C6'-C7'-C8' (blue).

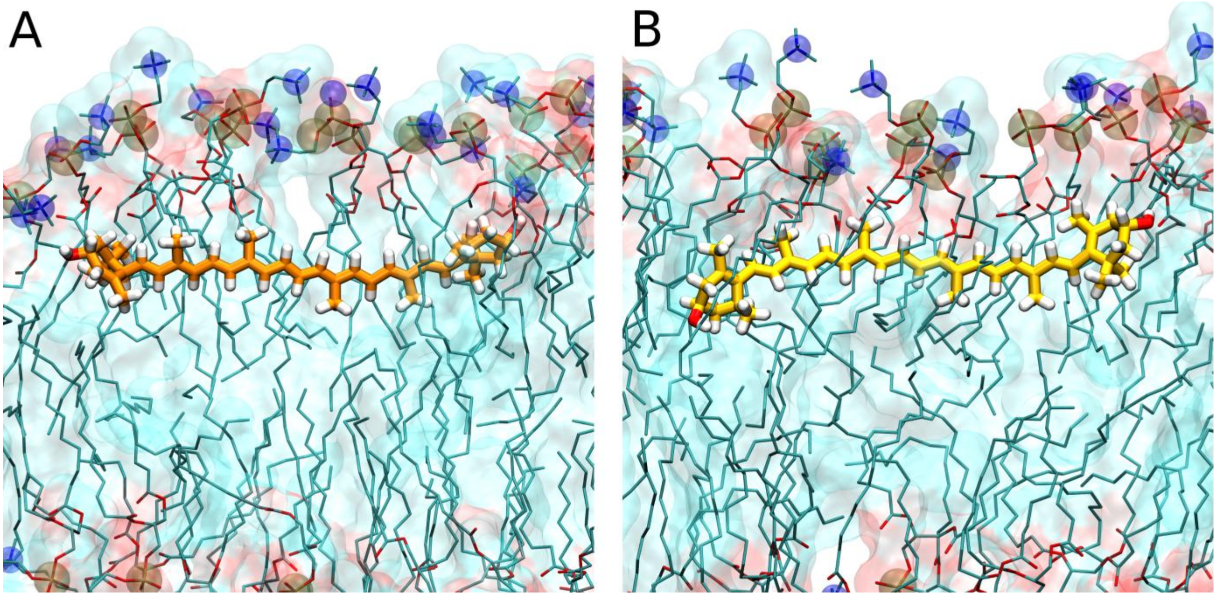

Figure S5. MD snapshots showing the representative arrangements of lutein (A) and zeaxanthin (B) in their horizontal orientation in the membrane. It can be seen that the ε-ring of lutein can flexibly rotate to incorporate into the polar region of the membrane, while the conformationally rigid β-ring of zeaxanthin is necessarily exposed to the hydrophobic core.

Table S1. Dihedral force field parameters for the rotation around the C6’-C7’ bond, obtained by fitting to a quantum mechanically calculated potential energy surface (full parametrization used for xanthophylls is provided in Tables S2-S4 and in the supplementary text file).

Dihedral definition	Force constant [kcal mol^{-1} deg^{-2}]	Periodicity	Phase shift
CG331 CG2D1 CG311 CG2DC1	1.393	3	0
HGA4 CG2DC1 CG311 CG2D1	1.414	1	0
HGA4 CG2DC1 CG311 CG2D1	0.734	3	0
HGA4 CG2DC1 CG311 CG301	0.228	3	0
HGA4 CG2DC1 CG311 CG301	0.077	1	180

Table S2. Topology of zeaxanthin and lutein.

Zeaxanthin topology			Bonds	
Atom name	Atom type	partial charge		
			C1	H1
C1	CG2DC1	-0.151	C1	C21
C2	CG2DC2	-0.149	C1	C2
H1	HGA4	0.150	C2	C3
H2	HGA4	0.150	C2	H2
C3	CG2DC2	-0.003	C3	C4
C4	CG331	-0.268	C3	C5
H3	HGA3	0.090	C4	H5
H4	HGA3	0.090	C4	H3
H5	HGA3	0.090	C4	H4
C5	CG2DC1	-0.150	C5	C6
C6	CG2DC1	-0.150	C5	H6
H6	HGA4	0.150	C6	H7
H7	HGA4	0.150	C6	C7
C7	CG2DC2	-0.149	C7	C8
C8	CG2DC2	-0.003	C7	H8
H8	HGA4	0.150	C8	C20
C9	CG2DC1	-0.149	C8	C9
C10	CG2DC1	-0.150	C9	C10
H9	HGA4	0.150	C9	H9
H10	HGA4	0.150	C10	H10
C11	CG2DC2	0.001	C10	C11
C12	CG301	0.010	C11	C12
C13	CG321	-0.191	C11	C16
C14	CG311	0.144	C12	C19
H11	HGA2	0.090	C12	C18
H12	HGA2	0.090	C12	C13
H13	HGA1	0.090	C13	H11
O1	OG311	-0.651	C13	H12
H14	HGP1	0.419	C13	C14
C15	CG321	-0.192	C14	C15
C16	CG2DC2	0.010	C14	O1
H15	HGA2	0.090	C14	H13
H16	HGA2	0.090	O1	H14
C17	CG331	-0.268	C15	C16
H17	HGA3	0.090	C15	H15
H18	HGA3	0.090	C15	H16
H19	HGA3	0.090	C16	C17
C18	CG331	-0.271	C17	H18
H20	HGA3	0.090	C17	H19
H21	HGA3	0.090	C17	H17
H22	HGA3	0.090	C18	H20
C19	CG331	-0.271	C18	H22
H23	HGA3	0.090	C18	H21
H24	HGA3	0.090	C19	H23
H25	HGA3	0.090	C19	H25
C20	CG331	-0.268	C19	H24
H26	HGA3	0.090	C20	H27
H27	HGA3	0.090	C20	H26
H28	HGA3	0.090	C20	H28
C21	CG2DC1	-0.151	C21	H29
C22	CG2DC2	-0.149	C21	C22
H29	HGA4	0.150	C22	C23
H30	HGA4	0.150	C22	H30
C23	CG2DC2	-0.003	C23	C40
C24	CG2DC1	-0.150	C23	C24
H31	HGA4	0.150	C24	C25
C25	CG2DC1	-0.150	C24	H31
C26	CG2DC2	-0.149	C25	H32
H32	HGA4	0.150	C25	C26
H33	HGA4	0.150	C26	C27
C27	CG2DC2	-0.003	C26	H33
C28	CG2DC1	-0.149	C27	C39
H34	HGA4	0.150	C27	C28
C29	CG2DC1	-0.150	C28	H34
C30	CG2DC2	0.001	C28	C29
H35	HGA4	0.150	C29	H35
C31	CG301	0.010	C29	C30
C32	CG321	-0.191	C30	C31
H36	HGA2	0.090	C30	C35
H37	HGA2	0.090	C31	C38
C33	CG311	0.144	C31	C37
O2	OG311	-0.651	C31	C32
H38	HGA1	0.090	C32	H37
H39	HGP1	0.419	C32	H36
C34	CG321	-0.192	C32	C33
C35	CG2DC2	0.010	C33	C34
H40	HGA2	0.090	C33	H38
H41	HGA2	0.090	C33	O2
C36	CG331	-0.268	O2	H39
H42	HGA3	0.090	C34	C35
H43	HGA3	0.090	C34	H40
H44	HGA3	0.090	C34	H41
C37	CG331	-0.271	C35	C36
H45	HGA3	0.090	C36	H42
H46	HGA3	0.090	C36	H43
H47	HGA3	0.090	C36	H44
C38	CG331	-0.271	C37	H45
H48	HGA3	0.090	C37	H46
H49	HGA3	0.090	C37	H47
H50	HGA3	0.090	C38	H50
C39	CG331	-0.268	C38	H48
H51	HGA3	0.090	C38	H49
H52	HGA3	0.090	C39	H52
H53	HGA3	0.090	C39	H53
C40	CG331	-0.268	C39	H51
H54	HGA3	0.090	C40	H54
H55	HGA3	0.090	C40	H56
H56	HGA3	0.090	C40	H55

Lutein topology			Bonds	
Atom name	Atom type	partial charge		
			C1	C2
C1	CG301	0.010	C1	C6
C2	CG321	-0.191	C1	C16
C3	CG311	0.144	C1	C17
C4	CG321	-0.192	C2	C3
C5	CG2DC1	0.010	C2	H1
C6	CG2DC1	0.001	C2	H2
C7	CG2DC2	-0.150	C3	C4
C8	CG2DC2	-0.149	C3	O1
C9	CG2DC1	-0.003	C3	H3
C10	CG2DC1	-0.149	C4	C5
C11	CG2DC2	-0.150	C4	H4
C12	CG2DC2	-0.150	C4	H5
C13	CG2DC1	-0.003	C5	C6
C14	CG2DC1	-0.149	C5	C18
C15	CG2DC2	-0.151	C6	C7
C16	CG331	-0.271	C7	C8
C17	CG331	-0.271	C7	H6
C18	CG331	-0.268	C8	C9
C19	CG331	-0.268	C8	H7
C20	CG331	-0.268	C9	C10
O1	OG311	-0.651	C9	C19
C21	CG301	0.023	C10	C11
C22	CG321	-0.194	C10	H8
C23	CG311	0.145	C11	C12
C24	CG2D1	-0.144	C11	H9
C25	CG2D1	-0.074	C12	C13
C26	CG311	-0.067	C12	H10
C27	CG2DC2	-0.100	C13	C14
C28	CG2DC2	-0.154	C13	C20
C29	CG2DC1	-0.002	C14	C15
C30	CG2DC1	-0.149	C14	H11
C31	CG2DC2	-0.150	C15	C35
C32	CG2DC2	-0.150	C15	H12
C33	CG2DC1	-0.003	C16	H13
C34	CG2DC1	-0.149	C16	H14
C35	CG2DC2	-0.151	C16	H15
C36	CG331	-0.274	C17	H16
C37	CG331	-0.274	C17	H17
C38	CG331	-0.270	C17	H18
C39	CG331	-0.268	C18	H19
C40	CG331	-0.268	C18	H20
O2	OG311	-0.650	C18	H21
H1	HGA2	0.090	C19	H22
H2	HGA2	0.090	C19	H23
H3	HGA1	0.090	C19	H24
H4	HGA2	0.090	C20	H25
H5	HGA2	0.090	C20	H26
H6	HGA4	0.150	C20	H27
H7	HGA4	0.150	O1	H28
H8	HGA4	0.150	C21	C22
H9	HGA4	0.150	C21	C26
H10	HGA4	0.150	C21	C36
H11	HGA4	0.150	C21	C37
H12	HGA4	0.150	C22	C23
H13	HGA3	0.090	C22	H29
H14	HGA3	0.090	C22	H30
H15	HGA3	0.090	C23	C24
H16	HGA3	0.090	C23	O2
H17	HGA3	0.090	C23	H31
H18	HGA3	0.090	C24	C25
H19	HGA3	0.090	C24	H32
H20	HGA3	0.090	C25	C26
H21	HGA3	0.090	C25	C38
H22	HGA3	0.090	C26	C27
H23	HGA3	0.090	C26	H33
H24	HGA3	0.090	C27	C28
H25	HGA3	0.090	C27	H34
H26	HGA3	0.090	C28	C29
H27	HGA3	0.090	C28	H35
H28	HGP1	0.419	C29	C30
H29	HGA2	0.090	C29	C39
H30	HGA2	0.090	C30	C31
H31	HGA1	0.090	C30	H36
H32	HGA4	0.150	C31	C32
H33	HGA1	0.090	C31	H37
H34	HGA4	0.150	C32	C33
H35	HGA4	0.150	C32	H38
H36	HGA4	0.150	C33	C34
H37	HGA4	0.150	C33	C40
H38	HGA4	0.150	C34	C35
H39	HGA4	0.150	C34	H39
H40	HGA4	0.150	C35	H40
H41	HGA3	0.090	C36	H41
H42	HGA3	0.090	C36	H42
H43	HGA3	0.090	C36	H43
H44	HGA3	0.090	C37	H44
H45	HGA3	0.090	C37	H45
H46	HGA3	0.090	C37	H46
H47	HGA3	0.090	C38	H47
H48	HGA3	0.090	C38	H48
H49	HGA3	0.090	C38	H49
H50	HGA3	0.090	C39	H50
H51	HGA3	0.090	C39	H51
H52	HGA3	0.090	C39	H52
H53	HGA3	0.090	C40	H53
H54	HGA3	0.090	C40	H54
H55	HGA3	0.090	C40	H55
H56	HGP1	0.413	O2	H56

Table S3. Bonded parameters for lutein and zeaxanthin.

BONDS

Atom types		Force constant [kcal mol^{-1} Å^{-2}]	Equilibrium length [Å]
G2DC1	CG301	365.00	1.5020
CG2DC2	CG301	365.00	1.5020
CG301	CG311	222.50	1.5000
CG301	CG321	222.50	1.5380
CG301	CG331	222.50	1.5380
CG311	CG321	222.50	1.5380
CG311	OG311	428.00	1.4200
CG311	HGA1	309.00	1.1110
CG2D1	CG311	365.00	1.5020
CG2DC2	CG311	365.00	1.5020
CG2D1	CG321	365.00	1.5020
CG2DC1	CG321	365.00	1.5020
CG2DC2	CG321	365.00	1.5020
CG321	HGA2	309.00	1.1110
CG2DC1	CG331	383.00	1.5040
CG2DC2	CG331	383.00	1.5040
CG2D1	CG2D1	440.00	1.3400
CG2D1	HGA4	360.50	1.1000
CG2DC1	CG2DC1	440.00	1.3400
CG2DC1	CG2DC2	300.00	1.4500
CG2DC1	HGA4	360.50	1.1000
CG2DC2	CG2DC2	440.00	1.3400
CG2DC2	HGA4	360.50	1.1000
OG311	HGP1	545.00	0.9600

ANGLES

Atom types			Force constant [kcal mol^{-1} deg^{-2}]	equilibrium angle [deg]
CG2DC1	CG2DC1	CG301	48.00	123.50
CG2DC2	CG2DC1	CG301	48.00	123.50
CG2DC1	CG2DC2	CG301	48.00	123.50
CG2DC2	CG2DC2	CG301	48.00	123.50
CG2DC1	CG301	CG321	32.00	112.20
CG2DC1	CG301	CG331	32.00	112.20
CG2DC2	CG301	CG321	32.00	112.20
CG2DC2	CG301	CG331	32.00	112.20
CG311	CG301	CG321	58.35	113.50
CG311	CG301	CG331	58.35	113.50
CG321	CG301	CG331	58.35	113.50
CG331	CG301	CG331	58.35	113.50
CG301	CG311	HGA1	34.60	110.10
CG301	CG321	HGA2	26.50	110.10
CG301	CG331	HGA3	33.43	110.10
CG2D1	CG311	CG301	32.00	112.20
CG2DC2	CG311	CG301	32.00	112.20
CG301	CG321	CG311	58.35	113.50
CG311	CG301	CG321	58.35	113.50
CG311	CG301	CG331	58.35	113.50
CG301	CG311	CG311	52.00	108.00
CG301	CG311	HGA1	34.60	110.10
CG321	CG311	CG321	58.35	113.50
CG321	CG311	OG311	75.70	110.00
CG321	CG311	HGA1	34.50	110.10
OG311	CG311	HGA1	45.90	108.89
CG2D1	CG321	CG311	32.00	112.20
CG311	CG321	HGA2	33.43	110.10
CG311	OG311	HGP1	50.00	106.00
CG2D1	CG2D1	CG311	48.00	123.50
CG311	CG2D1	CG331	48.00	123.50
CG311	CG2D1	HGA4	40.00	116.00
CG2DC2	CG2DC2	CG311	48.00	123.50
CG311	CG2DC2	HGA4	40.00	116.00
CG2D1	CG311	CG2DC2	125.00	108.00
CG2D1	CG311	CG321	32.00	112.20
CG2D1	CG311	OG311	75.70	110.10
CG2D1	CG311	HGA1	45.00	111.50
CG2DC2	CG311	HGA1	45.00	111.50
CG2DC1	CG321	CG311	32.00	112.20
CG321	CG2DC1	CG331	48.00	123.50
CG2DC2	CG2DC2	CG321	48.00	123.50
CG321	CG2DC2	CG331	48.00	123.50
CG2DC1	CG321	HGA2	45.00	111.50
CG2DC2	CG321	HGA2	45.00	111.50
HGA2	CG321	HGA2	35.50	109.00
CG2DC1	CG2DC1	CG331	48.00	123.50
CG2DC2	CG2DC1	CG331	48.00	113.00
CG2DC1	CG2DC2	CG331	48.00	113.00
CG2DC2	CG2DC2	CG331	48.00	123.50
CG331	CG2DC2	HGA4	42.00	117.50
CG2DC1	CG331	HGA3	42.00	111.50
CG2DC2	CG331	HGA3	42.00	111.50
CG2D1	CG2D1	HGA4	52.00	119.50
CG2DC1	CG2DC1	CG2DC2	48.00	123.00
CG2DC1	CG2DC1	HGA4	42.00	119.00
CG2DC2	CG2DC1	HGA4	42.00	118.00
CG2DC1	CG2DC2	CG2DC2	48.00	123.00
CG2DC1	CG2DC2	HGA4	42.00	118.00
CG2DC2	CG2DC2	HGA4	42.00	119.00

DIHEDRALS

Atom types				Force constant [kcal mol^{-1} deg^{-2}]	Periodicity	Phase shift
CG301	CG2DC1	CG2DC1	CG321	10.0000	2	180.00
CG301	CG2DC1	CG2DC1	CG331	10.0000	2	180.00
CG2DC1	CG2DC1	CG2DC2	CG301	0.9000	1	0.00
CG2DC1	CG2DC1	CG2DC2	CG301	2.1000	2	180.00
CG2DC1	CG2DC1	CG2DC2	CG301	0.2200	3	0.00
CG2DC1	CG2DC1	CG2DC2	CG301	0.2500	5	180.00
CG2DC1	CG2DC1	CG2DC2	CG301	0.1000	6	0.00
CG301	CG2DC1	CG2DC2	CG2DC2	0.9000	1	0.00
CG301	CG2DC1	CG2DC2	CG2DC2	2.1000	2	180.00
CG301	CG2DC1	CG2DC2	CG2DC2	0.2200	3	0.00
CG301	CG2DC1	CG2DC2	CG2DC2	0.2500	5	180.00
CG301	CG2DC1	CG2DC2	CG2DC2	0.1000	6	0.00
CG301	CG2DC1	CG2DC2	HGA4	1.0000	2	180.00
HGA4	CG2DC1	CG2DC2	CG301	1.0000	2	180.00
CG2DC1	CG2DC1	CG301	CG321	0.5000	2	0.00
CG2DC1	CG2DC1	CG301	CG321	0.3000	3	0.00
CG2DC1	CG2DC1	CG301	CG331	0.5000	2	0.00
CG2DC1	CG2DC1	CG301	CG331	0.4000	3	0.00
CG2DC2	CG2DC1	CG301	CG321	0.3000	3	0.00
CG2DC2	CG2DC1	CG301	CG331	0.3000	3	0.00
CG301	CG2DC2	CG2DC2	CG321	10.0000	2	180.00
CG301	CG2DC2	CG2DC2	CG331	10.0000	2	180.00
CG2DC1	CG2DC2	CG301	CG321	0.3000	3	0.00
CG2DC1	CG2DC2	CG301	CG331	0.3000	3	0.00
CG2DC2	CG2DC2	CG301	CG321	0.5000	2	0.00
CG2DC2	CG2DC2	CG301	CG321	0.3000	3	0.00
CG2DC2	CG2DC2	CG301	CG331	0.5000	2	0.00
CG2DC2	CG2DC2	CG301	CG331	0.4000	3	0.00
CG321	CG301	CG311	HGA1	0.1950	3	0.00
CG331	CG301	CG311	HGA1	0.1950	3	0.00
CG2DC1	CG301	CG321	HGA2	0.1900	3	0.00
CG2DC2	CG301	CG321	HGA2	0.1900	3	0.00
CG311	CG301	CG321	HGA2	0.1950	3	0.00
CG331	CG301	CG321	HGA2	0.1950	3	0.00
CG2DC1	CG301	CG331	HGA3	0.1600	3	0.00
CG2DC2	CG301	CG331	HGA3	0.1600	3	0.00
CG311	CG301	CG331	HGA3	0.1600	3	0.00
CG321	CG301	CG331	HGA3	0.1600	3	0.00
CG331	CG301	CG331	HGA3	0.1600	3	0.00
CG2D1	CG2D1	CG311	CG301	0.5000	1	180.00
CG2D1	CG2D1	CG311	CG301	1.3000	3	180.00
CG331	CG2D1	CG311	CG301	0.1900	3	0.00
CG2DC2	CG2DC2	CG311	CG301	0.5000	2	0.00
CG2DC2	CG2DC2	CG311	CG301	0.3000	3	0.00
HGA4	CG2DC2	CG311	CG301	0.2280	3	0.00
HGA4	CG2DC2	CG311	CG301	0.0770	1	180.00
CG321	CG301	CG311	CG2D1	0.2000	3	0.00
CG321	CG301	CG311	CG2DC2	0.2000	3	0.00
CG331	CG301	CG311	CG2D1	0.2000	3	0.00
CG331	CG301	CG311	CG2DC2	0.2000	3	0.00
CG2DC1	CG301	CG321	CG311	0.2000	3	0.00
CG311	CG301	CG321	CG311	0.2000	3	0.00
CG331	CG301	CG321	CG311	0.2000	3	0.00
CG2D1	CG311	CG321	CG301	0.1400	1	180.00
CG2D1	CG311	CG321	CG301	0.1700	2	0.00
CG2D1	CG311	CG321	CG301	0.0500	3	180.00
CG321	CG311	CG321	CG301	0.2000	3	0.00
OG311	CG311	CG321	CG301	2.0000	3	180.00
OG311	CG311	CG321	CG301	0.4000	5	0.00
OG311	CG311	CG321	CG301	0.8000	6	0.00
HGA1	CG311	CG321	CG301	0.1950	3	0.00
CG321	CG301	CG311	HGA1	0.1950	3	0.00
CG331	CG301	CG311	HGA1	0.1950	3	0.00
CG311	CG301	CG321	HGA2	0.1950	3	0.00
CG311	CG301	CG331	HGA3	0.1600	3	0.00
CG321	CG311	CG321	HGA2	0.1950	1	0.00
OG311	CG311	CG321	HGA2	0.1950	3	180.00
HGA1	CG311	CG321	CG2D1	0.2000	3	0.00
HGA1	CG311	CG321	HGA2	0.1950	3	0.00
CG321	CG311	OG311	HGP1	0.3000	1	0.00
CG321	CG311	OG311	HGP1	0.3000	3	0.00
HGA1	CG311	OG311	HGP1	0.0000	3	0.00
CG311	CG2D1	CG2D1	CG311	0.4500	1	180.00
CG311	CG2D1	CG2D1	CG311	8.5000	2	180.00
CG311	CG2D1	CG2D1	CG331	0.4500	1	180.00
CG311	CG2D1	CG2D1	CG331	8.5000	2	180.00
CG311	CG2D1	CG2D1	HGA4	1.0000	2	180.00
CG2D1	CG2D1	CG311	CG2DC2	1.0000	1	180.00
CG2D1	CG2D1	CG311	CG2DC2	0.1000	2	0.00
CG2D1	CG2D1	CG311	CG2DC2	0.3000	3	180.00
CG2D1	CG2D1	CG311	CG2DC2	0.2000	4	0.00
CG2D1	CG2D1	CG311	CG301	0.5000	1	180.00
CG2D1	CG2D1	CG311	CG301	1.3000	3	180.00
CG2D1	CG2D1	CG311	CG321	0.6000	1	180.00
CG2D1	CG2D1	CG311	OG311	1.9000	1	180.00
CG2D1	CG2D1	CG311	OG311	0.4000	2	180.00
CG2D1	CG2D1	CG311	OG311	0.6000	3	180.00

CG2D1	CG2D1	CG311	HGA1	0.3000	3	180.00
CG331	CG2D1	CG311	CG2DC2	1.3930	3	0.00
CG331	CG2D1	CG311	CG301	0.1900	3	0.00
CG331	CG2D1	CG311	HGA1	0.1900	3	0.00
HGA4	CG2D1	CG311	CG321	0.1200	3	0.00
HGA4	CG2D1	CG311	OG311	0.2000	3	0.00
HGA4	CG2D1	CG311	HGA1	0.0000	3	0.00
CG311	CG2D1	CG331	HGA3	0.1600	3	0.00
CG2DC1	CG2DC1	CG321	CG311	0.5000	2	0.00
CG2DC1	CG2DC1	CG321	CG311	0.3000	3	0.00
CG331	CG2DC1	CG321	CG311	0.1900	3	0.00
CG2DC1	CG2DC2	CG2DC2	CG311	0.5600	1	180.00
CG2DC1	CG2DC2	CG2DC2	CG311	7.0000	2	180.00
CG311	CG2DC2	CG2DC2	HGA4	5.2000	2	180.00
CG2DC2	CG2DC2	CG311	CG2D1	0.0000	3	180.00
CG2DC2	CG2DC2	CG311	CG301	0.5000	2	0.00
CG2DC2	CG2DC2	CG311	CG301	0.3000	3	0.00
CG2DC2	CG2DC2	CG311	HGA1	0.0300	3	0.00
HGA4	CG2DC2	CG311	CG2D1	1.4140	1	0.00
HGA4	CG2DC2	CG311	CG2D1	0.7340	3	0.00
HGA4	CG2DC2	CG311	CG301	0.2280	3	0.00
HGA4	CG2DC2	CG311	CG301	0.0770	1	180.00
HGA4	CG2DC2	CG311	HGA1	0.2000	3	0.00
CG321	CG301	CG311	CG2D1	0.2000	3	0.00
CG321	CG301	CG311	CG2DC2	0.2000	3	0.00
CG331	CG301	CG311	CG2D1	0.2000	3	0.00
CG331	CG301	CG311	CG2DC2	0.2000	3	0.00
CG2DC1	CG301	CG321	CG311	0.2000	3	0.00
CG311	CG301	CG321	CG311	0.2000	3	0.00
CG331	CG301	CG321	CG311	0.2000	3	0.00
CG2D1	CG311	CG321	CG301	0.1400	1	180.00
CG2D1	CG311	CG321	CG301	0.1700	2	0.00
CG2D1	CG311	CG321	CG301	0.0500	3	180.00
CG2D1	CG311	CG321	HGA2	0.1950	3	0.00
CG321	CG311	CG321	CG2DC1	0.2000	3	0.00
CG321	CG311	CG321	CG301	0.2000	3	0.00
OG311	CG311	CG321	CG2DC1	0.2000	3	0.00
OG311	CG311	CG321	CG301	2.0000	3	180.00
OG311	CG311	CG321	CG301	0.4000	5	0.00
OG311	CG311	CG321	CG301	0.8000	6	0.00
HGA1	CG311	CG321	CG2DC1	0.2000	3	0.00
HGA1	CG311	CG321	CG301	0.1950	3	0.00
CG2D1	CG311	OG311	HGP1	1.3000	1	0.00
CG2D1	CG311	OG311	HGP1	0.7000	2	0.00
CG2D1	CG311	OG311	HGP1	0.1400	3	0.00
CG2DC2	CG2DC1	CG2DC1	CG321	0.5600	1	180.00
CG2DC2	CG2DC1	CG2DC1	CG321	7.0000	2	180.00
CG2DC1	CG2DC1	CG321	HGA2	0.0300	3	0.00
CG331	CG2DC1	CG321	HGA2	0.1900	3	0.00
CG321	CG2DC1	CG331	HGA3	0.1600	3	0.00
CG2DC1	CG2DC2	CG2DC2	CG321	0.5600	1	180.00
CG2DC1	CG2DC2	CG2DC2	CG321	7.0000	2	180.00
CG301	CG2DC2	CG2DC2	CG321	10.0000	2	180.00
CG2DC2	CG2DC2	CG321	HGA2	0.0300	3	0.00
CG331	CG2DC2	CG321	HGA2	0.1900	3	0.00
CG321	CG2DC2	CG331	HGA3	0.1600	3	0.00
CG2DC2	CG2DC1	CG2DC1	CG331	0.5600	1	180.00
CG2DC2	CG2DC1	CG2DC1	CG331	7.0000	2	180.00
CG331	CG2DC1	CG2DC1	HGA4	5.2000	2	180.00
CG2DC1	CG2DC1	CG2DC2	CG331	1.1000	1	180.00
CG2DC1	CG2DC1	CG2DC2	CG331	0.7000	2	180.00
CG331	CG2DC1	CG2DC2	CG2DC2	1.1000	1	180.00
CG331	CG2DC1	CG2DC2	CG2DC2	0.7000	2	180.00
CG331	CG2DC1	CG2DC2	HGA4	1.0000	2	180.00
HGA4	CG2DC1	CG2DC2	CG331	1.0000	2	180.00
CG2DC1	CG2DC1	CG331	HGA3	0.3000	3	180.00
CG2DC2	CG2DC1	CG331	HGA3	0.3000	3	180.00
CG2DC1	CG2DC2	CG2DC2	CG331	0.5600	1	180.00
CG2DC1	CG2DC2	CG2DC2	CG331	7.0000	2	180.00
CG331	CG2DC2	CG2DC2	HGA4	5.2000	2	180.00
CG2DC1	CG2DC2	CG331	HGA3	0.3000	3	180.00
CG2DC2	CG2DC2	CG331	HGA3	0.3000	3	180.00
CG2DC2	CG2DC1	CG2DC1	CG2DC2	0.5600	1	180.00
CG2DC2	CG2DC1	CG2DC1	CG2DC2	7.0000	2	180.00
CG2DC2	CG2DC1	CG2DC1	HGA4	5.2000	2	180.00
HGA4	CG2DC1	CG2DC1	HGA4	5.2000	2	180.00
CG2DC1	CG2DC1	CG2DC2	CG2DC2	0.5000	1	0.00
CG2DC1	CG2DC1	CG2DC2	CG2DC2	2.0000	2	180.00
CG2DC1	CG2DC1	CG2DC2	CG2DC2	1.0000	3	0.00
CG2DC1	CG2DC1	CG2DC2	HGA4	1.0000	2	180.00
HGA4	CG2DC1	CG2DC2	CG2DC2	1.0000	2	180.00
HGA4	CG2DC1	CG2DC2	HGA4	0.0000	2	180.00
CG2DC1	CG2DC2	CG2DC2	CG2DC1	0.5600	1	180.00
CG2DC1	CG2DC2	CG2DC2	CG2DC1	7.0000	2	180.00
CG2DC1	CG2DC2	CG2DC2	HGA4	5.2000	2	180.00
HGA4	CG2DC2	CG2DC2	HGA4	5.2000	2	180.00

Table S4. Non-bonded parameters for lutein and zeaxanthin.

Nonbonded			
Atom type	cdiel	fshift	vatom
CG301	0.0	-0.0320	2.0000
CG311	0.0	-0.0320	2.0000
CG321	0.0	-0.0560	2.0100
CG331	0.0	-0.0780	2.0500
CG2D1	0.0	-0.0680	2.0900
CG2DC1	0.0	-0.0680	2.0900
CG2DC2	0.0	-0.0680	2.0900
OG311	0.0	-0.1921	1.7650
HGA1	0.0	-0.0450	1.3400
HGA2	0.0	-0.0350	1.3400
HGA3	0.0	-0.0240	1.3400
HGA4	0.0	-0.0310	1.2500
HGP1	0.0	-0.0460	0.2245
